# Independent associations of total and high molecular weight adiponectin with cardiometabolic risk and surrogate markers of enhanced early atherogenesis in black and white patients with rheumatoid arthritis: a cross-sectional study

**DOI:** 10.1186/ar4308

**Published:** 2013-09-20

**Authors:** Patrick H Dessein, Angela J Woodiwiss, Gavin R Norton, Linda Tsang, Ahmed Solomon

**Affiliations:** 1 Cardiovascular Pathophysiology and Genomics Research Unit, School of Physiology, Faculty of Health Sciences, University of the Witwatersrand, Johannesburg, South Africa; 2 Milpark Hospital, Johannesburg, South Africa; 3 Department of Rheumatology, Charlotte Maxeke Johannesburg Academic Hospital, Faculty of Health Sciences, University of the Witwatersrand, Johannesburg, South Africa

## Abstract

**Introduction:**

Whether adiponectin levels associate with atherogenesis in RA is uncertain. We examined the independent relationships of total and high molecular weight (HMW) adiponectin concentrations with cardiometabolic risk and surrogate markers of enhanced early atherogenesis in black and white patients with RA.

**Methods:**

We determined total and HMW adiponectin concentrations and those of endothelial activation molecules including soluble E-selectin, vascular cell adhesion molecule-1 (VCAM-1), intercellular adhesion molecule-1 (ICAM-1) and monocyte chemoattractant protein-1 (MCP-1), in 210 (119 black and 91 white) RA patients. Associations were determined in potential confounder and mediator adjusted mixed regression models.

**Results:**

Total and HMW adiponectin concentrations related similarly to metabolic risk factors and endothelial activation. In all patients, total and HMW adiponectin concentrations associated paradoxically with high systolic, diastolic and mean blood pressure (partial R = 0.155 to 0.241, *P* ≤0.03). Ethnic origin did not impact on these relationships (interaction *P* ≥0.09). Total and HMW adiponectin concentrations associated with those of glucose in white and black patients respectively (partial R = -0.304, *P* = 0.006 and -0.246, *P* = 0.01). In black but not white participants, total and HMW adiponectin concentrations also related favorably to lipid profiles (partial R = 0.292 to 0.360, *P* ≤0.003 for HDL cholesterol concentrations, -0.269 to -0.299, *P* ≤0.006 for triglyceride concentrations and -0.302 to -0.390, *P* ≤0.002 for total-HDL cholesterol ratio) and the number of metabolic risk factors (partial R = -0.210 to -0.238, *P* ≤0.03). In white but not black patients, total and HMW adiponectin concentrations associated paradoxically with overall endothelial activation as estimated by a standard z-score of endothelial activation molecule concentrations (partial R = 0.262, *P* = 0.01 and 0.252, *P* = 0.02); in the respective models, the extent of effect of total and HMW adiponectin concentrations on endothelial activation was larger in white compared to black participants (standardized β (SE) = 0.260 (0.107) versus -0.106 (0.107), *P* = 0.01 and 0.260 (0.120) versus -0.100 (0.111), *P* = 0.02). The HMW-total adiponectin ratio related inconsistently to metabolic risk factors and not to endothelial activation.

**Conclusion:**

In this study, total and HMW adiponectin concentrations associated with increased blood pressure parameters, and in white patients additionally with endothelial activation. The potential mechanism(s) underlying these paradoxical relationships between adiponectin concentrations and cardiovascular risk in RA merit further investigation.

## Introduction

Human adiponectin was identified in 1999 as the most abundant gene product in white adipose tissue [1]. Circulating concentrations of this adipokine are reduced in obesity [1,2]. Adiponectin decreases free fatty acid production and enhances insulin sensitivity [3] and its circulating concentrations associate with reduced plasma glucose and serum triglyceride levels and increased high density lipoprotein cholesterol concentrations, decreased blood pressure and a lower risk of type 2 diabetes [2,4-8]. These effects of adiponectin would be expected to translate into reduced cardiovascular disease risk. Besides, adiponectin additionally improves vascular health directly by mechanisms that include reduced endothelial activation through inhibition of nuclear factor *κ*B activation dependent endothelial adhesion molecule production [9] as well as the synthesis of endothelial monocyte chemoattractant protein-1 [10], a crucial molecule in early atherogenesis [11]. However, although an initial study revealed a reduced risk of myocardial infarction in relation to high adiponectin concentrations [12], a subsequent large prospective investigation and meta-analysis by Sattar and colleagues as reported in 2006, found no association with coronary heart disease risk [13]. Moreover, subsequent studies reported paradoxical positive relationships between adiponectin concentrations and cardiovascular disease risk in elderly subjects [14,15], patients with heart failure [14] or prevalent cardiovascular disease [16] and black Americans [17]. While among the different isoforms of adiponectin, it is particularly high molecular weight (HMW) adiponectin that confers the potential antidiabetic [18] and vascular protective activities [19] of adiponectin in the general population, a potential association with incident coronary heart disease was also not confirmed [20].

Adiponectin further modulates inflammatory and immune responses and was shown to be involved in the pathogenesis of rheumatoid arthritis (RA) [21-23]. Indeed, adiponectin induces gene expression and protein synthesis of many pro-inflammatory and pro-destructive molecules in several effector cells that participate in the pathophysiology of RA [24-27].

Our knowledge on the association of adiponectin levels with cardiovascular disease in RA is currently more limited [28-34]. Notably in this context, the presence of autoimmunity can alter the relationship between adipokines and cardiovascular disease risk [35-37]. Thus, Hahn and colleagues found that leptin administration enhanced pro-inflammatory high density lipoprotein scores as well as atherosclerosis in lupus prone but not non-immune mice [35]. Leptin concentrations also associated with cardiovascular risk in patients with lupus [36]. In addition, we recently documented that RA impacts on the relationships of total adiponectin concentrations with both lipid profiles and blood pressure among black Africans with RA [37]. Nevertheless, it remains unknown whether this finding represents either an ethnicity or disease specific effect among patients with RA. Importantly in the present context, genetic determinants of adiponectin levels [38] and adiponectin-cardiovascular disease relations in the general population differ by ethnic grouping [17,39].

In the present investigation, we examined the impact of population grouping on independent total and HMW adiponectin concentration-metabolic cardiovascular risk factor relationships and whether adiponectin levels associate with surrogate markers of enhanced early atherogenesis including soluble E-selectin, vascular cell adhesion molecule-1 (VCAM-1), intercellular adhesion molecule-1 (ICAM-1) and monocyte chemoattractant protein-1 (MCP-1) [40-47], in both black and white patients.

## Methods

### Patients

The present investigation was conducted according to the principles outlined in the Helsinki declaration. The Committee for Research on Human Subjects of the University of Witwatersrand approved the protocol (approval number: M06-07-33 in RA subjects). Participants gave informed, written consent. The study design was previously described [48-51]. Briefly, 210 African patients (119 black and 91 white) that met the 1987 American College of Rheumatology and 2010 American College of Rheumatology/European League Against Rheumatism criteria for RA [52,53] were enrolled at the Charlotte Maxeke Johannesburg Academic Hospital and Milpark Hospital [48-51]. All invited participants agreed to participate. Data were missing in fewer than 5% of any of the recorded characteristics.

Data on previously diagnosed established cardiovascular disease were derived by hospital record review.

### Assessments

We recorded demographic features and smoking status. Height, weight and waist and hip circumference were measured using standard approaches. The body mass index (BMI) was calculated and abdominal obesity and fat distribution were estimated by waist circumference and waist-hip ratio, respectively [48]. We recorded disease duration and rheumatoid factor status. Disease activity was assessed by the Disease Activity Score in 28 joints (DAS28) [54]. C-reactive protein concentrations were determined using immunoturbidimetric methods. Standard laboratory blood tests of renal and liver function, hematological parameters, lipids and glucose were performed. The glomerular filtration rate was estimated using the Modification of Diet in Renal Disease equation [55]. Cardiovascular drugs included antihypertensive agents and glucose and lipid lowering drugs.

Among metabolic risk factors, hypertension was defined as an average systolic blood pressure ≥140 or/and diastolic blood pressure ≥90 mmHg or/and current use of antihypertensive medications. Dyslipidemia was diagnosed when the atherogenic index, that is, the cholesterol:high density lipoprotein (HDL) cholesterol ratio was >4 and proatherogenic non-HDL cholesterol concentrations were calculated by subtracting HDL cholesterol from total cholesterol concentrations [48-51,56-59]. Diabetes was identified as the use of glucose lowering agents or a fasting plasma glucose ≥7 mmol/l. We calculated the number of metabolic risk factors using the National Cholesterol Education Program defined metabolic syndrome (MetS) definitions for MetS blood pressure, HDL cholesterol, triglycerides and glucose [60].

We measured endothelial activation molecule concentrations including those of soluble E-selectin, VCAM-1, ICAM-1 and MCP-1 using a solid-phase sandwich enzyme linked immunosorbant assay (Quantikine®HS, R & D Systems, Inc., Minneapolis, MN, USA). Their lower detection limits were 0.009 ng/l, 0.6 ng/l, 0.096 ng/l and 5.0 pg/ml, respectively; their inter- and intra-assay coefficients of variation were 7.9 and 5.8, 7.0 and 3.1, 5.5 and 4.6 and 5.7 and 5.8, respectively.

Total and HMW adiponectin concentrations were measured using solid-phase sandwich enzyme-linked immunosorbant assays (ELISA) (Quantikine®HS, R&D Systems, Inc.). Their lower detection limits were 0.246 and 0.195 ng/ml respectively. The inter- and intra-assay coefficients of variation were 6.5 and 3.5% for total and 8.5 and 3.0% for high molecular weight adiponectin, respectively.

### Data management and analysis

Dichotomous variables are expressed as proportions or percentages and continuous variables as mean (SD), or median (interquartile range) when non-normally distributed. Non-normally distributed characteristics were also logarithmically transformed prior to their inclusion in multivariable statistical analysis. An endothelial activation score was employed to provide a summary measure of endothelial activation and was calculated from SD (z) scores as follows: [z (selectin) + z (VCAM-1) + z (ICAM-1) + z (MCP-1)] [61].

Disparities in baseline characteristics, cardiovascular drug use, metabolic risk factors, endothelial activation molecule concentrations and adiponectin variables between African black and white patients with RA were assessed using the Student’s *t*-test, Mann–Whitney U test and univariate logistic regression analysis as appropriate.

The associations of age, sex and population grouping with adiponectin variables were assessed by entering the respective characteristics together in single mixed regression models. Associations of other baseline characteristics with adiponectin variables were evaluated in models with adjustment for all three demographic characteristics.

The independent relations of adiponectin variables with metabolic risk factors and early endothelial activation were assessed in demographic characteristic, glomerular filtration rate and waist circumference (potential confounders or/and determinants identified in previous analysis) and cardiovascular drug use adjusted mixed linear regression models. The impact of population grouping on the relations of adiponectin variables with metabolic risk factors and endothelial activation was assessed by adding an interaction term (population grouping (white = 1; black = 2) x adiponectin variable) to the models, and in stratified analysis, that is, in black and white participants separately.

Statistical computations were made using the GB Stat™ program (Dynamic Microsystems, Inc, Silver Spring, MD, USA) and SAS software, version 9.1 (The SAS Institute, Cary, NC, USA).

## Results

### Baseline characteristics, cardiovascular drug use, metabolic risk factors, endothelial activation and adiponectin variables in African black and white patients with RA

Table 1 shows that as compared to their white counterparts, black patients were more often women, smoked less frequently, had a higher BMI but lower waist-hip ratio, higher DAS28, C-reactive protein concentrations and glomerular filtration rate and used more conventional disease-modifying antirheumatic drugs (DMARDs) but no biologic agents; they experienced more prevalent hypertension, higher blood pressure values and differences in some of the recorded individual lipid variables but not in their cholesterol-HDL cholesterol and triglycerides-HDL cholesterol ratios, and used lipid lowering agents less often and oral glucose lowering drugs more frequently. The number of metabolic risk factors was larger in black compared with white patients. E-selectin concentrations were higher and those of ICAM-1 and MCP-1 lower in black compared to white participants; black patients had lower HMW adiponectin concentrations and, consequently, their HMW-total adiponectin ratio was numerically lower.

**Table 1:** Baseline characteristics, cardiovascular drug use, metabolic risk factors, surrogate markers of enhanced early atherogenesis and adiponectin variables in African black and white patients with rheumatoid arthritis

**Characteristics**	**Black (n = 119)**	**White (n = 91)**	***P***
Baseline characteristic			
Age, years	55.8 (10.2)	58.6 (10.9)	0.06
Female (%)	**89.1**	**76.7**	**0.02**
Smoking	**3.4**	**11.1**	**0.04**
Body mass index, kg/m^2^	**29.3 (6.6)**	**25.7 (4.7)**	**<0.0001**
Waist circumference, cm	93.3 (13.4)	90.2 (12.7)	0.08
Waist-hip ratio	**0.85 (0.80 to 0.90)**	**0.87 (0.82 to 0.93)**	**0.03**
RA duration, years	12.8 (9.2)	14.1 (9.3)	0.3
Rheumatoid factor positive (%)	77.3	76.4	0.8
DAS28	**4.2 (1.3)**	**3.6 (1.6)**	**0.007**
C-reactive protein, mg/ml	**7.0 (4.0 to 14.5)**	**4.1 (1.9 to 11.8)**	**0.007**
Glomerular filtration rate, ml/min/1.73 m^2^ Conventional disease modifying agents use	**105 (1)**	**89 (1)**	**0.0001**
Any (%)	100	100	1.0
Number	**2.5 (1.0)**	**2.2 (0.09)**	**0.02**
Methotrexate (%)	**90.8**	**79.1**	**0.02**
Chloroquine (%)	**79.8**	**55.0**	**0.0002**
Leflunomide (%)	**20.2**	**38.5**	**0.004**
Sulphasalazine (%)	**24.1**	**12.1**	**0.03**
Azathioprine (%)	16.8	11.0	0.2
Tetracycline (%)	10.1	13.2	0.5
Cyclophosphamide (%)	5.9	1.1	0.1
Penicillamine (%)	4.2	2.2	0.4
Tumor necrosis-α inhibitor (%)	0.0	8.8	…
Rituximab (%)	0.0	1.5	…
Prednisone (%)	1.7	3.3	0.5
Cardiovascular drug use			
Antihypertensive agents (%)	54.6	44.4	0.2
Oral glucose lowering agents (%)	**13.5**	**4.4**	**0.04**
Insulin (%)	0.8	2.2	0.4
Statin (%)	**19.4**	**38.9**	**0.002**
Ezetimibe (%)	0.0	2.2	…
Metabolic risk factors			
Hypertension (%)	**72.3**	**49.5**	**0.0008**
Systolic blood pressure, mmHG	**140 (25)**	**130 (17)**	**0.0004**
Diastolic blood pressure, mmHG	**86 (15)**	**80 (9)**	**0.0005**
Mean blood pressure, mmHg	**104 (17)**	**113 (13)**	**0.0002**
Cholesterol-HDL cholesterol ration >4 (%)	21.7	14.8	0.2
Total cholesterol, mmol/l	**4.7 (0.9)**	**5.1 (1.1)**	**0.004**
HDL cholesterol, mmol/l	1.5 (1.3 to 1.8)	1.6 (1.3 to 2.0)	0.07
Cholesterol-HDL cholesterol ratio	3.2 (1.1)	3.2 (1.0)	0.80
LDL cholesterol, mmol/l	**2.6 (0.8)**	**2.8 (0.9)**	**0.03**
Non HDL cholesterol, mmol/l	**3.1 (0.9)**	**3.4 (1.0)**	**0.05**
Triglycerides, mmol/l	1.0 (0.7 to 1.3)	1.0 (0.9 to 1.4)	0.4
Triglycerides-HDL cholesterol ratio	0.67 (0.46 to 1.10)	0.64 (0.46 to 0.93)	0.6
Diabetes (%)	16.0	7.8	0.08
Glucose, mmol/l	4.9 (4.5 to 5.4)	4.7 (4.4 to 5.1)	0.1
Metabolic risk factors, number*	**1.4 (0.9)**	**1.0 (0.8)**	**0.005**
Endothelial activation			
E-selectin, ng/ml	**42.23 (19.78)**	**36.05 (17.20)**	**0.02**
VCAM-1, ng/ml	841.84 (696.33 to 1071.75)	791.01 (641.33 to 1033.44)	0.3
ICAM-1, ng/ml	**238.10 (170.92 to 314.52)**	**309.19 (256.51 to 384.74)**	**<0.0001**
MCP-1, pg/ml	**349.31 (224.75 to 665.07)**	**460.12 (329.71 to 681.86)**	**0.01**
Endothelial activation score	-0.25 (2.35)	0.32 (2.35)	0.09
Adiponectin variables			
Total adiponectin, ng/ml	7.41 (4.62 to 11.56)	7.25 (5.31 to 12.83)	0.4
HMW adiponectin, ng/ml	**2.65 (1.54 to 5.53)**	**3.82 (2.10 to 6.00)**	**0.05**
HMW-total adiponectin ratio	0.44 (0.20)	0.50 (0.27)	0.07

Results are expressed as mean (SD) or median (interquartile range) unless indicated otherwise. Significant disparities are shown in bold. *Includes MetS defined reduced high density lipoprotein cholesterol and elevated triglycerides, blood pressure and glucose criteria. DAS28, Disease activity score in 28 joints; HDL, High-density lipoprotein; HMW, High molecular weight; ICAM, Intercellular adhesion molecule; LDL, Low-density lipoprotein; MCP, Monocyte chemoattractant protein; RA, Rheumatoid arthritis; VCAM, Vascular cell adhesion molecule.

Total and high molecular weight adiponectin concentrations were highly correlated in all, black and white patients (R = 0.617 (*P* <0.0001), R = 0.801 (*P* <0.0001) and R = 0.478 (*P* <0.0001), respectively).

Only seven patients had previously diagnosed established cardiovascular disease that included one myocardial infarction (white), five cerebrovascular accidents (four white and one black) and one peripheral vascular disease (white).

### Associations between baseline recorded characteristics and adiponectin variables in patients with RA

As given in Table 2, in confounder adjusted analysis, age associated with HMW adiponectin concentrations, female gender with those of both total and HMW adiponectin concentrations and black ethnicity with low HMW-total adiponectin ratios. Among the anthropometric measures, BMI and, to a larger extent, waist circumference associated with low total and HMW adiponectin concentrations. An inverse relationship between glomerular filtration rate and total adiponectin concentrations approached significance. In contrast to the findings in a recently reported investigation [34] that was performed among early untreated patients with RA, disease activity was unrelated to adiponectin concentrations in those with treated established disease. Smoking status was not associated with adiponectin concentrations and also not related to endothelial activation (data not shown).

**Table 2:** Associations between baseline characteristics and total* and high-molecular weight adiponectin concentrations* and high molecular weight-total adiponectin ratios in patients with rheumatoid arthritis

	**Total**		**HMW**		**HMW-total**	
	**Adiponectin**		**Adiponectin**		**Adiponectin**	
**Characteristic**	**Partial R**	***P***	**Partial R**	***P***	**Partial R**	***P***
Age	0.082	0.2	**0.154**	**0.02**	0.115	0.09
Female	**0.163**	**0.01**	**0.160**	**0.02**	0.080	0.2
Black ethnicity	-0.088	0.2	-0.036	0.6	**-0.137**	**0.04**
Smoking	-0.090	0.7	-0.009	0.9	-0.055	0.4
Body mass index*	**-0.167**	**0.01**	**-0.136**	**0.05**	0.037	0.6
Waist circumference	**-0.217**	**0.001**	**-0.219**	**0.001**	-0.052	0.4
Waist-hip ratio*	-0.074	0.3	-0.115	0.1	-0.092	0.1
RA duration	0.019	0.7	-0.010	0.9	-0.032	0.6
RF positive	-0.024	0.7	-0.023	0.7	-0.017	0.8
DAS28	-0.077	0.3	0.043	0.5	0.081	0.3
C-reactive protein*	-0.083	0.2	0.018	0.8	0.068	0.3
GFR	-0.119	0.09	0.024	0.7	0.087	0.2

Associations were determined in demographic characteristic adjusted mixed linear regression models. Significant disparities are shown in bold. *Log transformed; DAS28, Disease activity score in 28 joints; GFR, Glomerular filtration rate; RA, Rheumatoid arthritis; RF, Rheumatoid factor.

In separate models in which age, sex, glomerular filtration rate, cardiovascular drug use and waist circumference were adjusted for, black ethnicity was not related to both total and HMW adiponectin concentrations (*P* = 0.9 for both) but associated with low HMW-total adiponectin concentration ratios (*P* = 0.05). Further adjustment for hypertension, diabetes and established cardiovascular disease did not alter these results (*P* = 0.9, 0.9 and 0.06 for black ethnicity-total and HMW adiponectin concentrations and -HMW-total adiponectin ratio relations respectively). These results were also similar in separate models in which age, sex, waist circumference, glomerular filtration rate and the number of metabolic risk factors (see Table 1) were entered as potential confounders or mediators (*P* = 0.7, 0.5 and 0.06 for black ethnicity-total and high molecular adiponectin concentrations and -HMW-total adiponectin ratio relations, respectively).

### Independent relations of adiponectin variables with metabolic risk factors and surrogate markers of enhanced early atherogenesis in patients with RA

Tables 3 and 4 show the demographic characteristic, glomerular filtration rate, cardiovascular drug use and waist circumference adjusted relations of adiponectin variables with metabolic risk factors and endothelial activation molecules in all patients. As given in Table 3, in all patients, total adiponectin concentrations were independently related to high systolic, diastolic and mean blood pressure and low total, low density lipoprotein (LDL) and non-HDL cholesterol and triglyceride concentrations, and low total-HDL cholesterol and triglycerides-HDL cholesterol ratios. Population grouping impacted on the total adiponectin-HDL cholesterol concentration, -total-HDL cholesterol ratio, -non-HDL cholesterol and -triglyceride concentration and triglyceride-HDL cholesterol ratio, -number of metabolic risk factors and -VCAM-1 concentration and -endothelial activation score relations. In stratified analysis, total adiponectin concentrations associated significantly with high blood pressure values as well favorable lipid parameters and low number of metabolic risk factors in black but not white patients; total adiponectin concentrations related to low glucose concentrations and large VCAM-1 and MCP-1 concentrations as well as large endothelial activation score in white but not black participants.

**Table 3:** Independent relations of total adiponectin concentrations* with cardiometabolic risk and surrogate markers of enhanced early atherogenesis in African black and white patients with rheumatoid arthritis

			**Patients**					
	**Interaction**		**All (n = 210)**		**Black (n = 119)**		**White (n = 91)**	
**Characteristic**	**Partial R**	***P***	**Partial R**	***P***	**Partial R**	***P***	**Partial R**	***P***
Metabolic risk factor								
Systolic blood pressure	0.039	0.6	**0.232**	**0.001**	**0.304**	**0.001**	0.157	0.1
Diastolic blood pressure	-0.032	0.7	**0.203**	**0.004**	**0.248**	**0.01**	0.200	0.07
Mean blood pressure	0.026	0.7	**0.241**	**0.008**	**0.298**	**0.002**	0.181	0.1
Total cholesterol	-0.139	0.06	**-0.164**	**0.02**	-0.138	0.1	-0.213	0.06
HDL cholesterol*	**0.201**	**0.005**	0.126	0.08	**0.360**	**0.002**	-0.170	0.1
Total-HDL cholesterol ratio	**-0.270**	**0.0002**	**-0.231**	**0.001**	**-0.390**	**<0.0001**	-0.027	0.9
LDL cholesterol	-0.131	0.07	**-0.158**	**0.03**	-0.178	0.08	-0.142	0.2
Non-HDL cholesterol	**-0.244**	**0.0007**	**-0.209**	**0.004**	**-0.292**	**0.003**	-0.132	0.2
Triglycerides*	**-0.204**	**0.005**	**-0.231**	**0.003**	**-0.299**	**0.002**	-0.060	0.5
Triglycerides-HDL cholesterol ratio*	**-0.254**	**0.0004**	**-0.221**	**0.002**	**-0.379**	**<0.0001**	0.045	0.7
Glucose*	-0.104	0.1	-0.126	0.08	-0.109	0.3	**-0.304**	**0.006**
Number of metabolic risk factors	**-0.244**	**0.0007**	-0.120	0.1	**-0.238**	**0.01**	0.047	0.6
Early atherogenesis								
Selectin	-0.068	0.3	-0.040	0.6	-0.062	0.5	-0.029	0.7
VCAM-1*	**-0.165**	**0.02**	0.122	0.09	-0.020	0.8	**0.262**	**0.01**
ICAM-1*	-0.109	0.1	0.012	0.9	-0.083	0.4	0.147	0.1
MCP-1*	-0.122	0.09	0.100	0.2	-0.001	1.0	**0.297**	**0.006**
Endothelial activation score	**-0.203**	**0.005**	0.080	0.3	-0.071	0.4	**0.262**	**0.01**

Relationships were determined in demographic characteristic, log glomerular filtration, cardiovascular drug use and waist circumference adjusted models. Significant associations are shown in bold. *Log transformed. HDL, High density lipoprotein; ICAM, Intercellular adhesion molecule; LDL, Low density lipoprotein; MCP, Monocyte chemoattractant protein; VCAM, Vascular cell adhesion molecule.

**Table 4:** Independent relations of high molecular weight adiponectin concentrations* with cardiometabolic risk and surrogate markers of enhanced early atherogenesis in African black and white patients with rheumatoid arthritis

			**Patients**					
	**Interaction**		**All (n = 210)**		**Black (n = 119)**		**White (n = 91)**	
**Characteristic**	**Partial R**	***P***	**Partial R**	***P***	**Partial R**	***P***	**Partial R**	***P***
Metabolic risk factor								
Systolic blood pressure	0.122	0.09	**0.217**	**0.002**	**0.304**	**0.001**	0.123	0.1
Diastolic blood pressure	0.022	0.7	**0.155**	**0.03**	**0.257**	**0.01**	0.143	0.2
Mean blood pressure	0.107	0.1	**0.216**	**0.002**	**0.298**	**0.002**	0.143	0.2
Total cholesterol	**-0.149**	**0.04**	-0.002	1.0	-0.095	0.3	0.074	0.5
HDL cholesterol*	**0.214**	**0.003**	**0.214**	**0.003**	**0.292**	**0.003**	0.180	0.1
Total-HDL cholesterol ratio	**-0.190**	**0.009**	**-0.196**	**0.006**	**-0.302**	**0.002**	-0.11**2**	0.3
LDL cholesterol	-0.119	0.1	-0.036	0.6	-0.121	0.2	0.018	0.8
Non-HDL cholesterol	**-0.190**	**0.009**	-0.094	0.1	**-0.221**	**0.03**	-0.002	1.0
Triglycerides*	**-0.212**	**0.009**	**-0.137**	**0.05**	**-0.269**	**0.006**	-0.003	0.9
Triglycerides-HDL cholesterol ratio*	**-0.211**	**0.003**	**-0.202**	**0.004**	**-0.329**	**0.0008**	0.094	0.4
Glucose*	**-0.215**	**0.003**	**-0.155**	**0.03**	**-0.246**	**0.01**	-0.143	0.2
Number of metabolic risk factors	**-0.193**	**0.007**	**-0.155**	**0.03**	**-0.210**	**0.03**	-0.148	0.2
Early atherogenesis								
Selectin	-0.044	0.5	-0.013	0.9	-0.051	0.6	-0.019	0.8
VCAM-1*	-0.079	0.2	0.128	0.08	-0.007	0.9	**0.240**	**0.03**
ICAM-1*	-0.129	0.08	0.103	0.1	-0.007	0.9	**0.286**	**0.009**
MCP-1*	-0.054	0.4	0.021	0.8	-0.011	0.9	0.153	0.1
Endothelial activation score	-0.123	0.07	0.098	0.1	-0.058	0.5	**0.252**	**0.02**

Relationships were determined in demographic characteristic, log glomerular filtration, cardiovascular drug use and waist circumference adjusted models. Significant associations are shown in bold. *Log transformed. HDL, High density lipoprotein; ICAM, Intercellular adhesion molecule; LDL, low density lipoprotein; MCP, Monocyte chemoattractant protein; VCAM, Vascular cell adhesion molecule.

As shown in Table 4, in all patients, HMW adiponectin concentrations were independently related to high systolic, diastolic and mean blood pressure and large HDL cholesterol concentrations, low total HDL cholesterol ratios and triglyceride concentrations, triglycerides-HDL cholesterol ratios, glucose concentrations and number of metabolic risk factors. Population grouping impacted on the HMW adiponectin-total cholesterol and -HDL cholesterol ratio, non-HDL cholesterol and triglyceride concentration, total-HDL cholesterol and triglycerides-HDL cholesterol ratio, glucose concentration and number of metabolic risk factors relations. In stratified analysis, HMW adiponectin concentrations associated significantly with high blood pressure values as well as favorable lipid parameters, low glucose concentrations and low number of metabolic risk factors in black but not white patients; HMW adiponectin concentrations related to large VCAM-1 and ICAM-1-1 concentrations as well as large endothelial activation score in white but not black participants.

As given in Additional file 1: Table S1, in all patients, HMW-total adiponectin ratio associated with high total cholesterol concentrations. Population grouping impacted on the HMW-total adiponectin ratio-total, LDL, non-HDL cholesterol and glucose concentration relations. In stratified analysis, HMW-total adiponectin ratio associated with low glucose concentrations in black but not white patients, and with large LDL-cholesterol concentrations in white but not back participants.

Because population grouping did not impact on total and HMW adiponectin-blood pressure relations but the respective associations were not significant in white patients in stratified analysis (Tables 3 and 4), we further compared the extent of effect of total and HMW adiponectin on blood pressure parameters between black and white patients, in the respective models. As shown in Table 5, the extent of effect of total and HMW adiponectin on blood pressure parameters was similar in black compared with white patients.

**Table 5:** Comparison of extent of effect of total and high molecular weight adiponectin concentrations on blood pressure and surrogate markers of enhanced early atherogenesis between black and white patients with rheumatoid arthritis, in mixed regression models

	**Standardized β (SE)**		
	**Black (n = 119)**	**White (n = 91)**	***P***
Total adiponectin* versus			
Systolic blood pressure	0.321 (0.099)	0.146 (0.103)	0.2
Diastolic blood pressure	0.257 (0.099)	0.176 (0.096)	0.5
Mean blood pressure	0.309 (0.098)	0.166 (0.101)	0.3
VCAM-1*	**-0.028 (0.109)**	**0.276 (0.110)**	**0.05**
MCP-1*	**-0.029 (0.112)**	**0.293 (0.105)**	**0.03**
Endothelial activation score	**-0.106 (0.107)**	**0.260 (0.107)**	**0.01**
HMW adiponectin* versus			
Systolic blood pressure	0.341 (0.101)	0.119 (0.107)	0.1
Diastolic blood pressure	0.207 (0.103)	0.148 (0.101)	0.6
Mean blood pressure	0.308 (0.089)	0.137 (0.106)	0.2
VCAM-1*	-0.015 (0.112)	0.257 (0.116)	0.09
ICAM-1*	**-0.086 (0.118)**	**0.292 (0.109)**	**0.02**
Endothelial activation score	**-0.100 (0.111)**	**0.260 (0.120)**	**0.02**

Significant associations are shown in bold. *Log transformed. ICAM, Intercellular adhesion molecule; MCP, Monocyte chemoattractant protein; VCAM, Vascular cell adhesion molecule.

By contrast and also as given in Table 5, except for the HMW-VCAM-1 relation (*P* = 0.09), the extent of effect of total and HMW adiponectin on surrogate markers of enhanced early atherogenesis in the respective models (Tables 2, 3 and 4) was larger in white compared to black patients.

Table 6 shows that the relations of total and HMW adiponectin concentrations with surrogate markers of enhanced early atherogenesis in white participants (Tables 3 and 4), is independent of not only potential confounders and/or determinants as identified in the present analysis (Table 2) but also of metabolic risk factors including the MetS defined HDL, triglycerides, blood pressure and glucose criteria [60] as well as smoking. Additional adjustment for C-reactive protein concentrations did also not materially alter these results (data not shown).

**Table 6:** Relations of total and high- molecular weight adiponectin concentrations with surrogate markers of enhanced early atherogenesis after further adjustment for the number of metabolic risk factors* and smoking in white patients with rheumatoid arthritis

**Association**	**Partial R**	***P***
Total adiponectin^†^ versus		
VCAM-1^†^	**0.272**	**0.01**
MCP-1^†^	**0.261**	**0.01**
Endothelial activation score	**0.235**	**0.03**
HMW adiponectin^†^ versus		
VCAM-1^†^	**0.235**	**0.03**
ICAM-1^†^	**0.259**	**0.01**
Endothelial activation score	**0.221**	**0.04**

Significant associations are shown in bold. *Metabolic risk factors that were forced into the models included MetS defined reduced high density lipoprotein cholesterol and elevated triglycerides, blood pressure and glucose. ^†^Log transformed. ICAM, Intercellular adhesion molecule; MCP, Monocyte chemoattractant protein; VCAM, Vascular cell adhesion molecule.

Finally, our findings on total and HMW adiponectin-blood pressure relations (Tables 3 and 4) were materially unaltered upon further adjustment for potentially confounding RA characteristics comprising disease duration (cumulative inflammation), DAS28, C-reactive protein concentrations, rheumatoid factor status (disease severity) and the employed number of conventional DMARDs and biologic agent and prednisone use [62-65] (data not shown).

## Discussion

In the present study, total and HMW adiponectin concentrations related to a similar extent to metabolic risk factors in RA, as was previously documented in the non-RA population [20]. We found two similarities and three disparities in adiponectin-cardiovascular risk relations in black compared to white patients with RA. The former comprised positive adiponectin-blood pressure variable and inverse adiponectin-glucose concentration associations, and the latter positive adiponectin-favorable lipid profile and overall number of metabolic risk factors relationships in black but not white patients as well as positive adiponectin-endothelial activation associations in white but not black participants.

Numerous experimental studies have documented the protective effects of adiponectin on obesity induced pathological conditions, including insulin resistance and enhanced atherogenesis [66]. Although an insulin sensitivity enhancing effect would be expected to reduce high blood pressure, direct effects of adiponectin on components of vascular tissue are considered more important in this context [66]. These comprise the activating effects of adiponectin on endothelial nitric oxide (NO) synthase and cyclooxygenase-2 leading to the production of NO and prostaglandin-I_2_ production, respectively, and the ability of adiponectin to promote macrophage polarization toward the anti-inflammatory phenotype, which results in reduced interleukin-6, tumor necrosis factor (TNF)-α and MCP-1 and increased arginase-1, interleukin-1 and macrophage *N*-acetyl-galactosamine specific lectin-1 by M1 and M2 macrophages, respectively [66]. Each of these processes improves endothelial function.

Patients with RA experience substantially increased risk for cardiovascular disease [67-72]. Over the recent past, paradoxical positive relations between adiponectin concentrations and cardiovascular risk were reported in non-RA populations at high risk of cardiovascular disease [14-17]. In this context, we recently also found for the first time a paradoxical positive association between total adiponectin concentrations and blood pressure parameters in African black RA but not non-RA subjects [37]. In the present investigation we assessed total and HMW adiponectin concentrations and comprehensively adjusted for potential mediating and confounding characteristics in our analysis. Our current finding of positive relations between both total and HMW adiponectin concentrations and each of three blood pressure variables that were further present to a statistically similar extent in black and white patients argues against our previous result [37] being spurious and supports the notion that these relationships may be RA specific. Additionally, we found paradoxical positive associations between total and HMW adiponectin concentrations and endothelial activation in white patients only and indeed, in the respective models, the extent of effect of adiponectin concentrations on surrogate markers of enhanced early atherogenesis was significantly larger in white compared to black study participants. Thus, this relationship was ethnic specific among patients with RA.

Importantly in this regard, not only do genetic loci associated with adiponectin levels differ in black compared with whites but also, in the Health ABC study, adiponectin concentrations associated independently with prevalent and incident coronary heart disease in black but not white non-RA Americans [17]. Taken together, the impact of ethnicity on the adiponectin-cardiovascular risk relation may be reversed in subjects with RA. Further, whereas white ethnicity associates with higher adiponectin concentrations among black and white non-RA Americans and this association is driven by visceral adiposity and metabolic risk factors [17,39], ethnicity was not related to adiponectin levels both before and after adjustment for waist circumference and metabolic risk factors in the present RA cohort. Congruent with our previous findings [37], our current results suggest that findings on adiponectin metabolism and its associations with cardiovascular risk as reported in the non-RA population should not be merely translated to the RA population.

Potential mechanisms underlying the reported paradoxical adiponectin-cardiovascular risk relations were recently comprehensively and elegantly proposed by Sattar [73]. Among six raised possibilities, an attractive option was that of reverse causality, whereby increased adiponectin concentrations represent a chronic or acute on chronic compensatory mechanism to counteract metabolic and vascular stress in subjects with acute coronary syndrome or heart failure [73]. Recommendations for investigations aimed at elucidating paradoxical adiponectin-cardiovascular risk relations were also given.

Total and HMW adiponectin concentrations were strongly interrelated in the present RA investigation but the HMW-total adiponectin ratio was inconsistently associated with metabolic risk factors. The HMW-total adiponectin ratio was also not related to endothelial activation.

The previously alluded to paradoxical relation between total adiponectin and coronary heart disease among non-RA black Americans was postulated to result from a decreased production of HMW relative to other adiponectin isoforms in this population group [17,39]. We indeed found that HMW adiponectin concentrations were lower in black compared to white patients and black ethnicity associated with a lower HMW-total adiponectin ratio in age and sex adjusted analysis. However, HMW adiponectin concentrations were also shown to be unrelated to incident coronary heart disease [20].

RA adipocytes and their surrounding macrophages produce adipokines that regulate systemic inflammation and the presence of a complex adipokine-mediated interaction among white adipose tissue, cardiovascular disease and chronic inflammatory disease like RA was previously proposed [74]. In this regard, in a series of white patients with severe RA undergoing anti-TNF-α infliximab therapy, high grade inflammation showed an independent negative correlation with circulating adiponectin concentrations whereas low adiponectin levels clustered with metabolic syndrome features including dyslipidemia and high plasma glucose concentrations that reportedly contribute to atherogenesis in RA [75]. However, adiponectin concentrations were not related to blood pressure in this study. In another series of non-diabetic and mostly non-obese patients with ankylosing spondylitis undergoing anti-TNF-α therapy, adiponectin concentrations related to insulin sensitivity and marginally to low BMI [76]. Taken together, these findings support a role of hypoadiponectinemia in cardiometabolic risk in chronic inflammatory rheumatic diseases. The present study shows that in RA, ethnicity and presumably genetic factors may modulate metabolic risk through mechanisms that include an effect mediated by adipokines.

Our study has strengths and limitations. We assessed both total and HMW adiponectin concentrations and the production of four endothelial activation molecules, which reportedly mediates the initial stages of atherosclerosis [40-43] and is inhibited by adiponectin [9-11]. Endothelial activation is markedly enhanced and associated with disease characteristics that further are strongly implicated in increased cardiovascular risk, and hence constitutes a promising tool in the elucidation of atherogenic mechanisms in RA [44-47]. Importantly, endothelial activation was not associated with disease activity variables in the present cohort of patients with established and treated RA [61]. The cross sectional design of the present investigation precludes drawing inferences on the direction of causality and our results need to be reproduced in a longitudinal study, preferably with the inclusion of cardiovascular event rates as an outcome variable. Alcohol intake and physical activity can associate with increased adiponectin concentrations [6]. Only 15.3% of patients in the present study consumed alcohol with a median of three units per week, alcohol consumption was not related to total and HMW adiponectin concentrations (*P* = 0.4 and 0.5) and its inclusion as an additional confounder in the models in Tables 3, 4, 5 and 6 did not alter our findings (data not shown). The same lack of relationships was also present with regard to physical activity [36] (data not shown). Finally, the relative role of genetic [38] versus environmental factors, including socioeconomic characteristics [6] in the ethnicity-adiponectin and adiponectin-cardiovascular risk relationships among patients of different population origin in RA, were not determined and merit further study.

## Conclusions

In this study, adiponectin concentrations related inversely to those of glucose, and in black patients to favorable lipid profiles. These relationships are similar to those reported in the non-RA population. However, adiponectin concentrations also independently associated with increased blood pressure parameters, and in white patients additionally with enhanced endothelial activation. The possible mechanism(s) underlying these paradoxical relationships together with the concurrent presence of beneficial associations merit further investigation in order to determine the potential role of adiponectin in cardiovascular disease as well as its concentrations in cardiovascular disease risk stratification in RA.

## Abbreviations

BMI: Body mass index; DAS28: Disease activity score in 28 joints; DMARDs: Disease-modifying antirheumatic drugs; ELISA: Enzyme-linked immunosorbant assay; GFR: Glomerular filtration rate; HDL: High density lipoprotein; HMW: High molecular weight; ICAM: Intercellular adhesion molecule; LDL: Low density lipoprotein; MCP: Monocyte chemoattractant protein; MetS: Metabolic syndrome; RA: Rheumatoid arthritis; RF: Rheumatoid factor; VCAM: Vascular cell adhesion molecule.

## Competing interests

The authors declared that they have no competing interests.

## Authors’ contributions

PHD contributed to the conception, design and data acquisition, performed the statistical analysis and drafted the manuscript. AJW and GRN contributed to the conception and design and analysis and interpretation of the data. LT contributed to the conception, design, data acquisition, management and analysis. AS contributed to the conception, design and data. All authors read and approved the final manuscript.

## Supplementary Material

Additional file 1: Table S1 Independent relations of high molecular weight-total adiponectin ratio with cardiometabolic risk and surrogate markers of enhanced early atherogenesis in African black and white patients with rheumatoid arthritis. Click here for file
