# Erratum: Independent associations of total and high molecular weight adiponectin with cardiometabolic risk and surrogate markers of enhanced early atherogenesis in black and white patients with rheumatoid arthritis: a cross-sectional study

**DOI:** 10.1186/s13075-014-0503-3

**Published:** 2014-12-20

**Authors:** Patrick H Dessein, Angela J Woodiwiss, Gavin R Norton, Linda Tsang, Ahmed Solomon

**Affiliations:** Cardiovascular Pathophysiology and Genomics Research Unit, School of Physiology, Faculty of Health Sciences, University of the Witwatersrand, Private Bag 3, Wits 2050, Johannesburg, South Africa; Milpark Hospital, P. O. Box 91155, Auckland Park 2006, Johannesburg, South Africa; Department of Rheumatology, Charlotte Maxeke Johannesburg Academic Hospital, Faculty of Health Sciences, University of the Witwatersrand, Private Bag 3, Wits 2050, Johannesburg, South Africa

After publication of our recent article [1], it has been brought to our attention that the adiponectin concentrations are mislabelled as nanograms/millilitre (ng/ml). The total and high molecular weight concentrations were measured in micrograms/millilitre (μg/ml).
