# Supplementary material for: Independent associations of total and high molecular weight adiponectin with cardiometabolic risk and surrogate markers of enhanced early atherogenesis in black and white patients with rheumatoid arthritis: a cross-sectional study
Source: Arthritis Res Ther. 2013 Sep 20;15(5):R128. doi: 10.1186/ar4308 (PMC3978563; doi:10.1186/ar4308)
Supplement: Additional file 1: Table S1 — Independent relations of high molecular weight-total adiponectin ratio with cardiometabolic risk and surrogate markers of enhanced early atherogenesis in African black and white patients with rheumatoid arthritis. [file ar4308-S1.doc]

**Additional file 1: Table S1 Independent relations of high molecular weight-total adiponectin ratio with cardiometabolic risk and surrogate markers of enhanced early atherogenesis in African black and white patients with rheumatoid arthritis**

|  | | | Patients | | | | | |
| --- | --- | --- | --- | --- | --- | --- | --- | --- |
|  | Interaction | | All (n=210) | | Black (n=119) | | White (n=91) | |
| Characteristic | Partial R | *P* | Partial R | *P* | Partial R | *P* | Partial R | *P* |
| Metabolic risk factor | | | | | | | | |
| Systolic blood pressure | -0.063 | 0.4 | 0.074 | 0.3 | 0.063 | 0.5 | 0.045 | 0.6 |
| Diastolic blood pressure | -0.125 | 0.09 | -0.037 | 0.6 | -0.058 | 0.5 | -0.037 | 0.7 |
| Mean blood pressure | -0.081 | 0.3 | 0.052 | 0.4 | -0.003 | 0.9 | 0.019 | 0.9 |
| Total cholesterol | **-0.228** | **0.001** | **0.162** | **0.03** | 0.035 | 0.7 | -0.168 | 0.1 |
| HDL cholesterol* | -0.013 | 0.9 | 0.091 | 0.2 | -0.003 | 0.9 | 0.211 | 0.06 |
| Total-HDL cholesterol ratio | -0.024 | 0.7 | 0.024 | 0.7 | 0.036 | 0.7 | 0.022 | 0.8 |
| LDL cholesterol | **-0.161** | **0.02** | 0.149 | 0.4 | 0.058 | 0.5 | **0.244** | **0.03** |
| Non-HDL cholesterol | **-0.157** | **0.03** | 0.111 | 0.1 | 0.055 | 0.5 | 0.190 | 0.09 |
| Triglycerides* | -0.050 | 0.5 | -0.035 | 0.6 | -0.042 | 0.6 | -0.024 | 0.8 |
| Triglycerides-HDL cholesterol ratio* | 0.020 | 0.8 | -0.068 | 0.3 | -0.032 | 0.7 | -0.131 | 0.2 |
| Glucose* | **-0.223** | **0.008** | -0.091 | 0.2 | **-0.195** | **0.04** | 0.102 | 0.4 |
| Number of metabolic risk factors | -0.015 | 0.8 | -0.011 | 0.9 | -0.029 | 0.7 | -0.073 | 0.5 |
| Early atherogenesis | | | | | | | | |
| Selectin | -0.003 | 09 | 0.017 | 0.8 | 0.042 | 0.6 | -0.039 | 0.7 |
| VCAM-1* | -0.020 | 0.3 | 0.070 | 0.3 | 0.055 | 0.6 | 0.061 | 0.6 |
| ICAM-1* | -0.054 | 0.5 | 0.097 | 0.2 | 0.042 | 0.6 | 0.132 | 0.2 |
| MCP-1* | 0.060 | 0.4 | -0.077 | 0.3 | -0.015 | 0.9 | -0.092 | 0.4 |
| Endothelial activation score | -0.012 | 0.9 | 0.043 | 0.5 | 0.052 | 0.6 | 0.022 | 0.8 |

Relationships were determined in demographic characteristic, log glomerular filtration, cardiovascular drug use and waist circumference adjusted models. Significant associations are shown in bold. *Log transformed. HDL, high density lipoprotein; LDL, low density lipoprotein; VCAM, vascular cell adhesion molecule; ICAM, intercellular adhesion molecule; MCP, monocyte chemoattractant protein.
